# Ethnobotanical Documentation, Phytochemical Screening, and Cytotoxicity Evaluation of Medicinal Plants Used to Manage Snakebite Envenomation in Mwingi West Subcounty, Kenya

**DOI:** 10.1155/2021/4167296

**Published:** 2021-09-27

**Authors:** Stella Kwamboka Mokua, James Mucunu Mbaria, Timothy Elias Maitho, Gervason Apiri Moriasi

**Affiliations:** ^1^Department of Public Health, Pharmacology and Toxicology, College of Veterinary and Agricultural Sciences, University of Nairobi, P.O. Box 29053-00625, Nairobi, Kenya; ^2^Department of Medical Biochemistry, College of Health Sciences, School of Medicine, Mount Kenya University, P.O. Box 342-01000, Thika, Kenya

## Abstract

Snakebite envenomation (SBE) is a life-threatening global public health problem affecting over 2.7 million persons annually, with a bigger burden lying in the developing world. Despite the successful management of SBE by antivenom therapy in conventional medicine, it is of low efficacy due to the diverse venom composition across snake types, which limits its usefulness. As a result, inhabitants of the sub-Sahara region, where SBE incidence is high, utilise medicinal plants as an alternative remedy for SBE. However, most plants have not been ethnobotanically documented and validated empirically and hence this study is needed. An ethnobotanical survey to document medicinal plants used to manage SBE in Migwani ward, Mwingi West Subcounty, Kitui County, was conducted between January and February, 2021. Ethnobotanical data were collected from 45 purposefully sampled respondents from Migwani ward using semistructured questionnaires, field walks, and oral interviews. In this study, 14 medicinal plants which are used to manage SBE were documented. Four plants with the highest Relative Frequency of Citation (RFC) (*Entada leptostachya* Harms-stem bark (0.58), *Senna singueana*-roots (0.53), *Securidaca longipendunculata*-roots (0.36), and *Strychnos henningsii*-stem bark (0.46)) were selected and extracted using water, methanol, and dichloromethane according to the standard procedures. Qualitative phytochemical analysis of the plant extracts and their cytotoxic effects on brine shrimp nauplii (brine shrimp lethality assay) was conducted according to the standard techniques. Qualitative phytochemical screening revealed the presence of anti-SBE-associated phytochemicals, such as alkaloids, saponins, tannins, phenols, and flavonoids, in the aqueous and methanolic extracts of the studied plant extracts. However, the tested phytochemicals were not detected in dichloromethane extracts of all the studied extracts. The anti-SBE effects of the documented plants could be attributable to these associated bioactive phytocompounds, which are synthesized by the studied plants and transfered to humans when consumed. Furthermore, the aqueous and methanolic extracts of *Entada leptostachya* and *Senna singueana* had high LC_50_ of >1000 *µ*g/ml and were considered noncytotoxic. However, *Securidaca longipendunculata* had an LC_50_ of <1000 *µ*g/ml and was considered slightly cytotoxic. Further empirical investigations to characterise the bioactive phytochemicals and their safety should be done.

## 1. Introduction

Snakebite is a neglected public health problem affecting over 2.7 million individuals annually, especially in those living in the most remote, underdeveloped, and marginalised tropical and subtropical regions of the world [1]. Snakebite envenoming/envenomation (SBE) accounts for over 138,000 deaths, leaving over 400,000 survivors with long-term psychological and physical disabilities [2]. Just like other poverty-associated diseases, there is insufficient public health policy frameworks, strategies, and investment in the affected regions, to sustainably reduce the medical and societal strain posed by SBE due to the lack of political goodwill and the demographic nature of the affected communities [2–4].

Subcutaneous or intramuscular injection of venom, via a snakebite, into the victim's body, elicits local and systemic toxic effects with profound sequela [5, 6]. Local effects associated with SBE include haemorrhage, oedema, myonecrosis, and extracellular matrix (ECM) degradation. Also, neurotoxicity, myotoxicity, cardiotoxicity, and hemotoxic syndrome are associated with systemic SBE sequelae [6].

Currently, antivenom therapy is the standard and arguably reliable strategy for averting the adverse effects caused by snake venom [7, 8]. However, despite the benefits of antivenom therapy, it evokes immediate hypersensitivity reactions, among other adverse effects, exhibits limited efficacy against local tissue damage, and suffers a stability deficit [9, 10]. Moreover, most antivenoms are ineffective due to geographic variation in venom composition and antigenic reactivity, attributable to the taxonomic diversity of venomous snake types [10, 11]. Besides, the high cost of antivenoms, especially in economically deprived settings, unavailability of enough antivenom stocks in various healthcare facilities, and inaccessibility of hospitals impede timely antivenom access, thereby leading to high morbidity and mortality rates [6, 8, 12].

In Kenya, approximately 15,000 cases of SBE are recorded annually, with 6.7 deaths per 100,000 persons, in the rural settings, accounting for ∼0.7% of all deaths [2]. Among the Kenyan counties, Kitui County has a comparatively higher SBE incidence caused by venomous snake species, including the puff adder (*Bitis arietans*), the black-necked cobra (*Naja nigricollis*), and the black mamba (*Dendroaspis polylepis*) [13]. This is attributable to the dry and hot climate of Kitui County, its geographic location, and the type of housing and agricultural activities which predispose inhabitants to snakebites [13]. Additionally, the unavailability of effective SBE treatment in most health facilities, especially those in rural areas, and the unaffordability of antivenom treatments by most of the population further complicate effective management of SBE in Kenya [14].

Due to the bottlenecks of the conventional antivenom therapy, compounded by low supplies in sub-Saharan Africa, various communities use medicinal plants in their traditional medicine practices to manage SBE complications [12]. Medicinal plants are critical for maintaining human health, especially SBEs in rural regions where it is not easy to obtain specific antivenoms [15]. Despite the longstanding usage of medicinal plants against SBE in traditional medicine, it has not been accorded sufficient attention in the scientific arena [12].

Research evidence has revealed the presence of various phytochemicals with antivenom properties, which present a viable alternative source of accessible, safe, and efficacious therapies for SBEs, especially in rural settings [16]. Moreover, plant-derived extracts successfully inhibit and reverse snake venom-induced inflammation, haemorrhage, myotoxicity, and neurotoxicity [17–22], and some others are used as prophylaxis against SBE complications in case of a snakebite [16].

Based on this background, the current study was conducted to identify and document medicinal plants used to manage venomous snakebites in Mwingi West Subcounty, Kenya, and evaluate their phytochemical composition and safety.

## 2. Materials and Methods

### 2.1. Study Area

**Figure 1:**
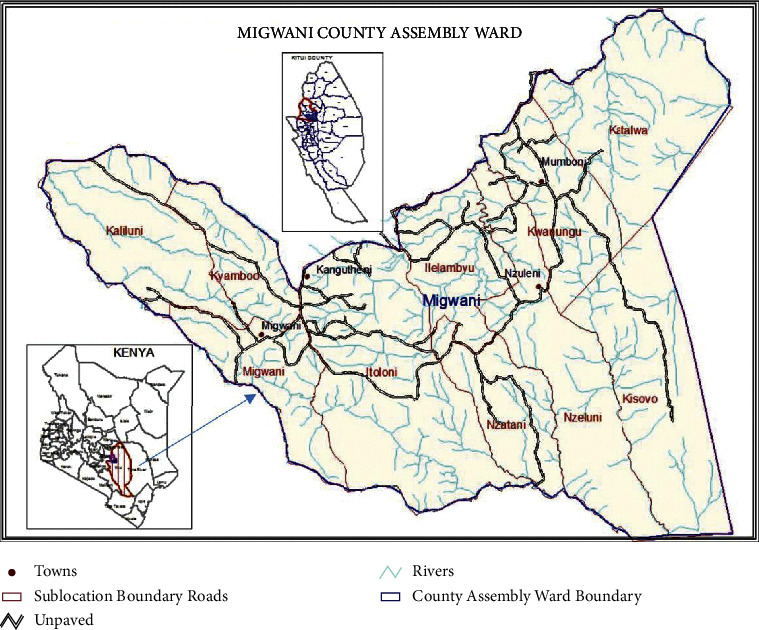
Map of Kenya showing the study site (Migwani ward) (adopted from the Kitui County integrated development plan 2018–2022 [23]).

Ethnobotanical documentation was carried out in Migwani ward, Mwingi West Subcounty, in Kitui County, Kenya (Figure 1), located about 49.7 km from Kitui town and 176.2 km from Nairobi city, lying along 35°1′49.044″N and 91°57′1.944″W. Migwani ward comprises six villages: Kyamboo/Kaliluni, Migwani/Itoloni, Nzatani/Ilalambyu, Nzeluni/Mung'alu, Kisovo, and Katalwa/Mumbuni [23, 24]. It is the most populated ward of Kitui County, with a population of 79,255 persons in 39,096 households, according to the 2019 national census report [25]. This region experiences a subhumid climate, hot and dry for almost the entire year, with an erratic and unreliable rainfall distribution. As a result, its lowest annual average temperature is 14°C, while the highest annual average temperature is 32°C. Most residents of the Migwani ward (67.3%) are small-scale farmers, with family members being the primary source of labour in the agricultural production system [23].

### 2.2. Ethnobotanical Data Collection and Analysis

The field survey was conducted between January and February 2021. The purposive sampling technique described by Palinkas et al. [26] was used to sample forty five participants aged between 20 and 80 years old who were knowledgeable about medicinal plants used to treat SBE in the study area. The inclusion criteria for selecting study respondents were herbalists and members of local community (based on their knowledge), natives who understood the local area, and local names of the plants. The initial participants were selected with the help of local leaders, local dwellers, and herbalists, who referred others through their existing networks within the study area. Saturation was reached when new data collection did not yield any new information on the medicinal plants used [26].

Following this, ethnobotanical data on medicinal plants used in the management of snakebites were collected through interviews (conducted either in the native language, Kamba, or Swahili, depending on the participants' preference), administration of semistructured questionnaires, and guided field walks to plant collection sites [27–30].

The collected data included the demographic information of the respondents (name, age, sex, practice specification, and level of education) and botanical information (local name of the plant(s) used, its source, availability of the plant(s), parts used, method of drug preparation and administration, any side effects, frequency of treatment, and duration of treatment). In addition, the respondents filled informed consent forms before taking part in the study.

### 2.3. Collection and Identification of Plants

Medicinal plants reportedly used in the management of SBE, as mentioned by the participants during the survey, were collected as voucher specimens by a team comprising herbalists and the researchers. Also, photographs of all the medicinal plants cited were taken to help during identification and for documentation. The plant specimens were identified at the East African Herbaria hosted at the National Museums of Kenya by botanists (Dr. Paul Kirika and Mr. Mathias M. Mbale), where reference numbers were assigned and voucher specimens deposited. Upon identification, plant materials were transported to the Department of Public Health, Pharmacology, and Toxicology Laboratories of the University of Nairobi for analysis.

### 2.4. Sample Preparation and Extraction

The plant materials were prepared according to the methods of Abubakar et al. [31] and Moriasi et al. [32] with slight modifications. Briefly, the collected plants' parts were washed with clean water, chopped into small pieces with a sharp knife, and dried at room temperature for two weeks. Upon drying, the plant materials were ground using an electric mill to a coarse powder, packed in khaki bags, and stored on a shelf awaiting extraction.

The extraction procedures described by Harborne et al. [33] and modified by Moriasi et al. [34] were followed in this study. Briefly, two hundred grams (200 g) of each powdered plant material was weighed and soaked in 1000 ml of 95% methanol and dichloromethane, respectively, at room temperature for 48 hours and regularly shaken using a mechanical shaker. The extracts were filtered twice, initially with cotton wool and later with a Whatman filter paper No. 1. The filtrates were evaporated to dryness at 40°C using a rotary evaporator/evaporating dish. The crude extracts were weighed and placed in a refrigerator at 4°C in sealed glass bottles until use.

To obtain the aqueous extracts, two hundred grams (200 g) of the powdered plant materials were weighed and transferred into conical flasks, and 1000 ml of distilled water was added and shaken. Afterwards, the flasks were placed in a hot water bath (70°C), heated for 2 hours, and filtered through cotton gauze and Whatman filter paper No. 1. The filtrates were transferred into freeze-drying flasks covered with dry carbon ice and acetone and freeze dried for 48 hours. The crude extracts were weighed and placed in the refrigerator at 4°C in sealed glass bottles until use.

### 2.5. Qualitative Phytochemical Screening of the Selected Plants

Qualitative phytochemical screening of the aqueous, methanolic, and dichloromethane extracts of the selected plants was performed using the methods described by Trease and Evans [35], Harborne et al. [33], and Moriasi et al. [36] with slight modifications to detect the presence or absence of various bioactive compounds. The phytochemicals tested include alkaloids, tannins, phenols, saponins, and flavonoids. Direct visual observations of the reactions' colouration profile or the formation of precipitates were done and used to appraise the presence or absence of respective phytochemicals in the study samples.

### 2.6. Evaluation of the Effects of the Selected Plant Extracts on Brine Shrimp Nauplii

In this study, the brine shrimp lethality assay technique of Meyer et al. [37] was used to determine the cytotoxic effects of the studied plant extracts to appraise their safety.

#### 2.6.1. Hatching of Brine Shrimp Nauplii

Brine shrimp eggs were commercially sourced from the Department of Public Health, Pharmacology and Toxicology, University of Nairobi. They were hatched in a rectangular box with two chambers having perforations. One chamber was dark, and a 40-watt electric bulb illuminated the other chamber. The box was filled with brine solution, after which 50 g of brine shrimp eggs was sprinkled with a spatula into the dark chamber of the box. Five grams of yeast was added as feed for the hatched nauplii. After 48 hours, the nauplii were collected from the illuminated chamber and used for the brine shrimp lethality test.

#### 2.6.2. Preparation of Plant Extracts

In this study, 0.1 g of the studied organic and aqueous plant extracts were weighed and dissolved in 1 ml of brine salt solution (38.5 g of marine salt in 1 litre distilled water) to make a stock concentration of 10,000 *µ*g/ml, which was then diluted serially.

#### 2.6.3. Brine Shrimp Lethality Assay

The assay was carried out to investigate the cytotoxicity of extracts of medicinal plants of Migwani according to the principle and protocol previously described by Meyer et al. [37], with slight modifications. Three dilutions were prepared by transferring 500 *µ*l, 50 *µ*l, and 5 *µ*l of plant extract *(Entada leptostachya, Senna singueana, Securidaca longipendunculata*, and *Strychnos henningsii*) into the set of five graduated tubes. Next, brine solution was added to obtain dilutions of 1000 *µ*g/ml, 100 *µ*g/ml, and 10 *µ*g/ml in five replicates. After that, ten (10) brine shrimp nauplii were transferred into each tube. Vincristine sulphate was used as a positive control, while brine solution was used as the negative control. Test tubes were left to settle at room temperature, and the surviving nauplii were counted after 24 hours. Probit regression analysis was performed to determine the median lethal concentration (LC_50_) of each studied plant extract in this assay.

### 2.7. Data Management, Analysis, and Reporting

Ethnobotanical and extract yield data were organised and summarised using Microsoft Office Excel 2013 software, where descriptive statistics were performed.

The Relative Frequency of Citation (RFC) criteria were used to determine popularly used plants to manage SBE in the study area. The relative frequency of citation (RFC) of plant species was calculated by dividing the frequency of citation (FC) (the number of respondents who cited a particular species) by the total number of respondents in the survey (*N* = 45). This RFC index ranges from zero (0) (when nobody refers to a plant as useful) to one (1) (when all respondents mention the species as useful). The following formula described by Vitalini et al. [38] was used to calculate the RFC index:

(1) $$\begin{array}{c} \text{RFC}=\frac{\text{FC}}{N}, \end{array}$$

where RFC is the relative frequency of citation, FC is the citation frequency, and *N* is the sample size (45 respondents).

Medicinal species with high RFC were selected for phytochemical analysis as per the recommendations of Rahman et al. [39].

The brine shrimp lethality assay data of the studied plant extracts were analysed using probit regression analysis. Mortalities were converted to probits and regressed against the log concentration of crude plant extracts (SPSS v20) [40, 41]. Meyers and Clarkson's criteria were used to infer the toxicity of substances tested in the brine shrimp lethality assay (LC_50_ values) [37, 42]. The findings of this study were presented in bar graphs (drawn using GraphPad Prism version 9.1.2) and tables.

## 3. Results

### 3.1. Ethnobotanical Documentation of Medicinal Plants Used to Manage SBE in the Study Area

#### 3.1.1. Sociodemographic Characteristics of the Study Participants

This study included 45 participants, aged between 20 and 80 years, who provided ethnobotanical information of medicinal plants used to manage SBE in the study area. In terms of gender, 58% of the respondents were male, while 42% were female. Most participants (53%) were aged between 41 and 60 years, followed by those aged between 20 and 40 years (31%), while those aged ≥61 years accounted for 16%. Only 2% of the respondents were formally employed, with the majority (67%) practising subsistence farming and other small-scale activities for livelihood.

The results further showed that 33% of the respondents had not acquired any formal education, 38% had a primary level, 18% had obtained a secondary level of education, and 11% had tertiary education. Most respondents (91%) were native local people, while 9% comprised herbalists. Ethnomedical knowledge was primarily acquired from family members and relatives (65%); 13% of the respondents acquired knowledge from herbalists, while 22% learnt from dreams/divine call/literature.

It was observed that most of the respondents (42%) had <5 years of ethnomedical experience, while those having 6–10 years of practice were 38%, and only 20% had practised for over ten years.  Table 1 presents the sociodemographic characteristics of the study participants.

#### 3.1.2. Ethnobotanical and Ethnomedical Information of Documented Plants and Frequency of Citation (FC and RFC)

Medicinal plants used to manage SBE in Migwani ward were documented, as well as their relevant information, as summarised in Table 2. In this study, 14 medicinal plants species belonging to 12 families were documented. The most represented family was Asteraceae with three plant species, while Capparaceae, Fabaceae, Burseraceae, Loganiaceae, Musaceae, Polygalaceae, Vitaceae, Solanaceae, Euphorbiaceae, Leguminacea, and Opiliaceae were represented by one plant species each (Table 2).

The most frequently cited plant species included *Entada leptostachya* (RFC = 0.56)*, Senna singueana* (RFC = 0.53)*, Strychnos henningsii* (RFC = 0.47), and *Securidaca longipendunculata* (RFC = 0.36), respectively (Table 2). Notably, all the documented plants were applied topically on the bitten site, with some being administered orally and topically (Table 2).

**Figure 2:**
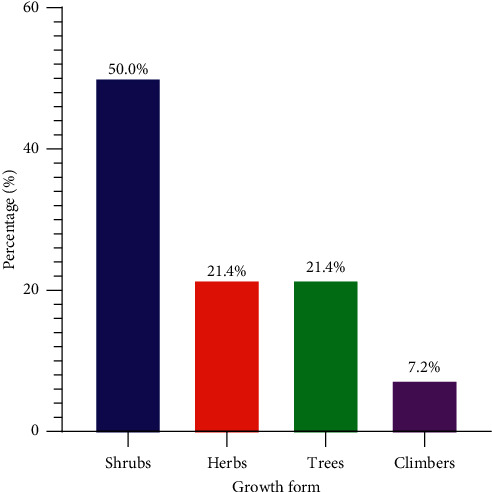
Growth forms of the documented plants.

The results also showed that most of the documented plants were shrubs (50%), followed by herbs (21.4%), trees (21.4%), and climbers (7.2%) (Table 2; Figure 2).

**Figure 3:**
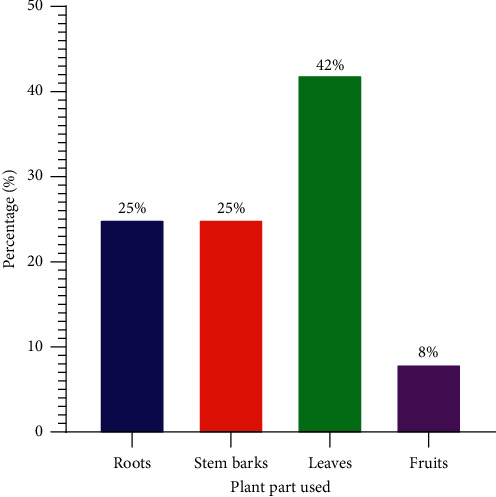
Plant part mostly used to prepare remedies for SBE in the study area.

The most used plant part(s) in the preparation of SBE remedies were the leaves (42%), roots (25%), stems/barks (25%), and fruit (8%), respectively (Table 2; Figure 3).

### 3.2. Extract Yields of the Selected Plants

Four medicinal plants with the highest RFC values were selected and extracted using water, methanol, and dichloromethane for qualitative phytochemical screening and brine shrimp lethality assay.

For the aqueous extracts, *Securidaca longipendunculata* had the highest yield (10%), followed by *Senna singueana* (5%) and *Strychnos henningsii* (5%), while *Entada leptostachya* had the lowest yield (4%) (Table 3). The highest percentage yield of the methanolic extracts was recorded by *Securidaca longipendunculata* (1.97%), followed by *Senna singueana* (1.43%), then *Entada leptostachya* (1.22%), and *Strychnos henningsii* (1.03%), respectively. On the other hand, the highest yield of dichloromethane extracted was obtained by *Senna singueana* (1.45%) followed by *Securidaca longipendunculata* (0.51%), *Entada leptostachya* (0.49%), and *Strychnos henningsii* (0.46%), respectively (Table 3).

### 3.3. Qualitative Phytochemical Composition of the Selected Plant Extracts

Qualitative phytochemical screening revealed the presence of alkaloids, phenols, and tannins in all the aqueous and methanolic extracts of the four studied plants (Table 4). Additionally, saponins and flavonoids were detected in all the aqueous and methanolic extracts, except in the methanolic extracts of *Senna singueana* and *Entada leptostachya* (Table 4). Conversely, alkaloids, phenols, flavonoids, tannins, and saponins were not detected in the dichloromethane extracts of all the studied plants (Table 4).

### 3.4. Cytotoxic Effects of the Aqueous, Methanolic, and Dichloromethane Extracts of the Studied Plants

The results revealed that all the aqueous extracts of the studied plants have high LC_50_ values (>1000 *µ*g/ml) except that of *Securidaca longipendunculata,* which posited an LC_50_ value of 170.66 *µ*g/ml (Table 5). Similarly, the methanolic extracts of all the studied plants had high LC_50_ values (>1000 *µ*g/ml), except *Securidaca longipendunculata,* which had an LC_50_ value of 293.97 *µ*g/ml (Table 5). Besides, no LC_50_ values were predicted for all the dichloromethane extracts of the selected plants, as no nauplii mortalities were recorded in the respective setups. Overall, the positive control drug (Vincristine) posited the lowest LC_50_ value of 4.06 *µ*g/ml in this study (Table 5).

## 4. Discussion

Herbal medicine plays a significant role in treating diverse diseases, especially in rural settings of less developed countries. A recent report by the WHO indicate that over 80% of the world's human population rely on medicinal plants for their primary healthcare needs [43]. The increased popularity of herbal medicines is attributable to their easy accessibility, affordability, and presumed safety compared to conventional medicines [44–47]. However, despite the longstanding usage of medicinal plants in traditional medicine, only a handful have been scientifically investigated. One of the hindrances to the appraisal of medicinal plants' potency and possible development is the lack of baseline ethnomedical information to spur empirical studies.

The management of SBE using medicinal plants has been practised since antiquity in many ethnic communities, especially those in rural settings [48]. However, in most traditions, ethnomedical knowledge, including the traditional management of SBE, is undocumented and often passed across generations by word of the mouth to trusted family members or relatives [49–52]. However, there is a high propensity to lose this critical information, especially if the family members are not interested and not appropriately documented [53]. As a result, ethnomedical documentation is an important undertaking for heritage, conservational strategies, and the advancement of research.

The current study's findings revealed that most respondents (65%) acquired ethnomedical knowledge through their family members and relatives. This finding corroborates that of Nadembega et al. [54], who asserted that traditional medicinal knowledge on plants is passed from generation to generation verbally to family members. However, most people who could inherit this art are often younger and disinterested, as they view it as archaic. As a result, valuable ethnomedical information is lost, especially when the bearer dies and no record of such information is available.

In the current study, shrub species were commonly used to manage SBE in Migwani ward, the study area. Perhaps, this could be attributed to their relatively high resistance to drought conditions experienced in the study area, hence leading to their unlimited availability throughout the year [55]. Besides, medicinal plants are mostly found on hills (Kea), valleys (Ikoo), rocky surfaces, and roadsides in the study area, thereby depicting their availability and accessibility. Furthermore, studies have shown that the abundance and availability of herbaceous plants in natural habitats such as forests largely influence their exploitation for medicinal purposes [54].

The most widely used plant part(s) in preparing SBE remedies were the leaves, perhaps due to their ease of harvest and availability in large quantities, compared to other plant parts [38]. Moreover, previous studies have indicated that the preference of leaves in traditional medicine to other parts is due to their perceived rich host of bioactive ingredients, such as alkaloids and tannins, associated with photosynthesis [56]. Furthermore, leaves produce and accumulate most phytochemical amalgams due to their involvement in photosynthesis [57]. Indeed, most phytochemicals possess pharmacologic activities, which are thought to confer therapeutic potency [34, 36, 58].

Notably, some respondents mentioned using single plant parts or blending many plant parts to thwart SBE complications. These findings are consistent with previous studies, which report the use of various plant parts to mitigate ailments [39, 59]. This practice could potentially be due to the synergistic effects of the combined plant parts, which produce amplified efficacy and in a short time, hence being helpful in cases of SBE [39].

The most common herbal preparation methods included infusions, poultices, tinctures, decoctions, and powders administered orally, topically, or both to avert SBE [60, 61]. It was observed that water was the primary medium for preparing most remedies, and additives like honey, cow milk, and sheep soup were added to enhance taste and palatability [62–65]. It suggests that the administration of herbals through different routes could increase the bioavailability of the drug's bioactive constituents and counter SBE sequelae.

It was notable that the mode of drug administration and treatment type depended on the type of snake, the age of the victim, and the presence of any other preexisting conditions. This implies that the respondents were knowledgeable about the basic pharmacologic principles of their medicines' activity. Besides, it was evident that the study participants understood the dangers of a drug overdose and indicated that they use mutton soup, cow's milk, honey, activated charcoal, and water as antidotes. However, the herbalists claimed the potency of their herbal formulations would reduce if they revealed some specific adjuvants they added. Moreover, they argued that unique offerings or rituals ought to be performed to reveal some of these adjuvants with the promise of utmost secrecy. This caveat is a tool employed by herbalists to protect their ethnomedical [66–68], thus hindering knowledge sharing, especially in this study.

The relative frequency of citation (RFC) was used to determine the most used medicinal plant for managing SBE in the study area. The RFC index indicates the reliability and accuracy of the collected information, as it reveals the medicinal plants best known or with a long history of use by most of the participants [69]. Our study revealed that *Entada leptostachya, Senna singueana, Strychnos henningsii,* and *Securidaca longipendunculata* had the highest RFC, indicating they were commonly utilised among the locals to manage SBE. As a result, these plants were selected and screened for their qualitative phytochemical composition and cytotoxic effects on brine shrimp nauplii to lay a framework for further characterisation and development of bioactive components that can be used as therapies for SBE.

Upon extraction, the yields of the studied plants' extracts were varied according to the solvent used. The variations were attributed to the different polarity indices of the solvents, which solubilise and extract amalgams of corresponding polarity [70]. Previously, Moriasi et al. [32] highlighted that polar solvents such as methanol and water extract antioxidant-associated phytochemicals, which possess diverse pharmacologic activities. Owing to the profound usage of *E. leptostachya, S. singueana, S. henningsii,* and *S. longipendunculata* to manage SBE in the study area, their pharmacologic efficacy could be due to the presence of polar phytocompounds, such as phenols and flavonoids [71]. Besides, the absence of certain phytochemicals in one sample and their presence in the others can be attributed to the plant's various physiological and biosynthetic reactions and the agroecological conditions of the study area [32, 72]. Additionally, the absence of the tested phytochemicals in the dichloromethane extracts of the studied plants could be attributed to the low polarity of the solvent, which hindered their extraction [70].

Previous studies indicate that various phytochemicals with protein binding properties, such as flavonoids, polyphenols, saponins, tannins, and alkaloids, bind to toxic venom proteins, thereby inactivating them [73]. Furthermore, flavonoids have been shown to inhibit phospholipases *A*_2_, an essential component of snake venoms [74]. Thus, the presence of flavonoids in all crude plant extracts may confirm their use in SBE management. Additionally, flavonoids, phenols, tannins, and alkaloids have been shown to act as antidotes to snake venoms, with the ability to reverse the deleterious effects of SBE [75, 76]. Therefore, the studied plant extracts are a valuable reservoir of bioactive compounds of pharmacological significance, which warrant further investigations.

In the present study, the brine shrimp lethality assay technique of Meyers et al. [37] was adopted to appraise the studied plant extracts' cytotoxicity. A drug agent or chemical that kills the exposed brine shrimp nauplii is considered a cytotoxic agent in this assay. Furthermore, median lethal concentration (LC_50_) values are widely used to evaluate the cytotoxic efficacy of drugs and chemicals in bioassays, whereby low values indicate high cytotoxic efficacy. According to Meyer's cytotoxicity classification criteria, plant extracts with LC_50_ < 1000 *µ*g/ml are considered toxic, while those with LC_50_ > 1000 *µ*g/ml are considered nontoxic, hence safe [37]. Additionally, Clarkson's toxicity criteria classify plant extracts as nontoxic (LC_50_ > 1000 *µ*g/ml), slightly toxic (LC_50_ = 500–999 *µ*g/ml), moderately toxic/toxic (LC_50_ = 99–499 *µ*g/ml), and highly toxic (LC_50_ = 0–100 *µ*g/ml), respectively [42].

Accordingly, based on Meyer's and Clarkson's criteria, the aqueous, methanolic, and dichloromethane extracts of *E. leptostachya, S. singueana,* and *S. henningsii* were nontoxic and safe to brine shrimp nauplii since their LC_50_ values were higher than 1000 *µ*g/ml. Therefore, the safety of these plants was attributed to the absence or low concentrations of toxicity-associated phytocompounds. Conversely, the aqueous and methanolic extracts of *S. longipendunculata* were moderately toxic, which calls for caution when used for SBE management. However, these extracts can be used as cytotoxic agents in appropriate settings. Nevertheless, further toxicological investigations should be performed to establish the toxicity profile and safety of the studied plant extracts.

## 5. Conclusions and Recommendations

The study revealed that fourteen medicinal plants were used to manage SBE in Migwani ward, Mwingi-West Subcounty, Kitui County, Kenya, and *Entada leptostachya, Strychnos henningsii, Securidaca longipendunculata,* and *Senna singueana* were most frequently cited. The most used plants against SBE in Migwani ward, West Mwingi Subcounty (*Entada leptostachya, Strychnos henningsii, Securidaca longipendunculata,* and *Senna singueana*) contain phytochemicals associated with snake antivenom. Three of the aqueous, methanolic, and dichloromethane extracts of the commonly used plants against SBE in Migwani ward, West Mwingi Subcounty (*Entada leptostachya, Strychnos henningsii,* and *Senna singueana*) are noncytotoxic and safe. In contrast, the aqueous and methanolic extracts of *Securidaca longipendunculata* are moderately toxic to brine shrimp nauplii.

Based on this study's findings, further empirical investigations of the studied plants and their extracts, especially to determine their potential to avert SBE in *in vivo* models, should be conducted. Besides, further phytochemical analyses to identify the specific bioactive molecules and their mode(s) of pharmacologic activity against SBE should be done. Finally, further toxicological investigations involving the studied plant extracts should be performed to establish the toxicological profiles and safety.

## Figures and Tables

**Table 1:** Sociodemographic characteristics of the study respondents.

Variable	Category	*N*	% *N*
Sex	Male	26	58
	Female	19	42

Age (years)	20–40	14	31
	41–60	24	53
	61–80	7	16

Education	Primary	17	38
	Secondary	8	18
	Tertiary	5	11
	None	15	33

Practise specifications	Herbalist	4	9
	Local people	41	91

Source of income	Employment	1	2
	Business	14	31
	Others	30	67

Experience (years)	0–5	19	42
	6–10	17	38
	>10	9	20

Source of knowledge	Relatives	29	65
	Herbalist	6	13
	Others	10	22

*N* = number of participants (sample size); % *N* = percentage of participants.

**Table 2:** Medicinals used to manage SBE in the study area.

Plant species (V/N)	Common name (local name)	Family	Growth form	Part(s) used	Preparation and dosage	Mode of administration	FC	RFC	
*Entada leptostachya* Harms. (NMK/BOT/CTX/5/4)	Entada/Sikidy (“*Mwaitha*”)	Fabaceae	Shrub	Stem, bark	Stem crushed, sap squeezed and applied directly to the wound.	Topical	26	0.56	
*Senna singueana* (Delile.) Lock. (NMK/BOT/CTX/1/4)	Winter cassia/sticky pod (“*Mukengeka*”*/*“*Mukengeta*”)	Leguminosae	Shrub	Leaves, roots	Roots dried in the sun, crushed into a fine powder and applied or mixed with sheep soup, one full glass drunk daily for five days. Leaf's infusion of the plant is drunk as an antidote for puff adder bites.	Topical Oral	24	0.53	
*Strychnos henningsii* Gilg. (NMK/BOT/CTX/5/6)	Walking stick/Panda's strychnos (“*Muteta*”)	Loganiaceae	Tree	Leaves, stem, bark, roots	Fresh roots can be chewed and swallowed to expel poison by vomiting. Leaves may be cooked with water or sheep soup of original intestines and taken twice daily quarter a glass.	Oral Topical	21	0.47	
*Securidaca longipendunculata* (NMK/BOT/CTX/5/1)	Violet plant (“*Munguuka*”)	Polygalaceae	Tree	Roots, leaves, barks	Soak the dried powdered root bark part in water, take thrice a day, or apply leave paste.	Topical or oral	16	0.36	
*Solanum incanum* (NMK/BOT/CTX/1/1)	Thorn apple (“*Kikondu*”*/*“*Mutongu*”)	Solanaceae	Shrub	Fruits, leaves	The stems or fruits are cut into small pieces, dried in the sun, pounded, and powder applied, or sap from the fruit may be applied directly.	Topical	11	0.24	
*Cissus rotundifolia* (Forsk.) Vahl. (NMK/BOT/CTX/1/2)	Peruvian grape ivy (“*Itulu*”)	Vitaceae	Shrub	Leaves	Sap from pounded leave is applied directly onto the wound four times for 10–14 days.	Topical	8	0.18	
*Musa x paradisiaca* L. (NMK/BOT/CTX/5/7)	Edible banana (“*Mathangu ma maiu*”)	Musaceae	Tree	Leaves, stem	Sap squeezed out of leaves and stem and applied immediately onto the snakebite site reducing swelling and pain.	Topical	5	0.11	
*Tagetes minuta* L. (NMK/BOT/CTX/1/6)	Mexican marigold (“*Muvangi*”)	Asteraceae	Herb	Leaves	Leaves are crushed or chewed rubbed into snakebite as an antidote.	Topical	4	0.09	
*Commiphora sp.* (NMK/BOT/CTX/5/5)	Commiphora/corkwood (“*Ithangu*”)	Burseraceae	Shrub	Leaves, fruit	The milky exudates from unripe fruits can be applied. Leaves are crushed. Infusion is taken orally half a cup once a day for three days. Leaves pounded applied directly.	Topical	2	0.04	
*Ricinus communis* L. (NMK/BOT/CTX/1/3)	Castor oil plant (“*Kyaiki*”*/*“*Kivaiki*”)	Euphorbiaceae	Shrub	Leaves	Fresh young leaves are pounded and tied to the snakebite for six hours to accelerate healing. The plant is cultivated at the homestead since it has a strong smell that causes discomfort or disorientation to snakes.	Topical	2	0.04	
*Boscia salicifolia* L. (NMK/BOT/CTX/5/2)	Willow-leaved shepherd's tree (“*Ithangana*”)	Capparaceae	Shrub	Barks, roots	Roots and barks burned into charcoal, crushed into fine powder, and applied twice for six days.	Topical	1	0.02	
*Kleinia abyssinica* A. Berger. (NMK/BOT/CTX/5/3)	Klenia (“*Ngondu ya* “*Kimani*”)	Asteraceae	Herb	Roots	Roots pounded and soaked in water and infusion drunk two glasses twice a day until the wound heals or is applied on the wound.	Topical oral	1	0.02	
*Gutenbergia cordifolia* Benth ex. Oliv. (NMK/BOT/CTX/5/8)	Gutenbergia (“*Ithungululu*”)	Asteraceae	Herb	Leave	Leaf's sun-dried burned ash is rubbed on the site daily for five days.	Topical	1	0.02	
*Opilia amentacea* Roxb. (NMK/BOT/CTX/1/5)	Opilia (“*Mutonga*”)	Opiliaceae	Climber	Roots	Roots cut into pieces, sun dried, then crushed into powder mixed with crushed snake teeth and applied for 7–10 days.	Topical	1	0.02	

V/N = voucher number; FC = frequency of citation; RFC = relative frequency of citation.

**Table 3:** Yields of the aqueous, methanolic, and dichloromethane extracts of the selected plants.

Plant and part extracted	Percentage yield		
	Aqueous extracts	Methanolic extracts	Dichloromethane extracts
*Entada leptostachya* (stem bark)	4	1.22	0.49
*Senna singueana* (root)	5	1.43	1.45
*Securidaca longipendunculata* (root)	10	1.97	0.51
*Strychnos henningsii* (stem bark)	5	1.03	0.46

**Table 4:** Qualitative phytochemical composition of the aqueous, methanolic, and dichloromethane extracts of the studied plants.

Phytochemical	*Entada leptostachya* stem bark			*Senna singueana* (Delile) roots			*Securidaca longipendunculata* roots			*Strychnos henningsii* Gilg stem bark		
	Aq.	Me.	Dc.	Aq.	Me.	Dc.	Aq.	Me.	Dc.	Aq.	Me.	Dc.
Alkaloids	+	+	−	+	+	−	+	+	−	+	+	−
Flavonoids	+	−	−	+	+	−	+	+	−	+	+	−
Saponins	+	+	−	+	−	−	+	+	−	+	+	−
Tannins	+	+	−	+	+	−	+	+	−	+	+	−
Phenols	+	+	−	+	+	−	+	+	−	+	+	−

*Note.* +: present; −: absent; Aq.: aqueous extract; Me.: methanolic extract; Dc.: dichloromethane extract.

**Table 5:** Cytotoxic effects of the aqueous, methanolic, and dichloromethane extracts of the selected plants.

Drug	LC_50_ (*µ*g/ml)		
	Aq.	Me.	Dc.
*E. leptostachya*	5789.69^#∗^	16108.21^#∗^	ND
*S. singueana*	24995.60^#∗^	230149.13^#∗^	ND
*S. longipendunculata*	170.66^##∗∗^	293.93^##∗∗^	ND
*S. henningsii*	1288.55^#∗^	2180.37^#∗^	ND
Vincristine sulphate	4.06^##∗∗∗^		

The superscript notations ^#^ and ^##^ represent noncytotoxic and cytotoxic, respectively, based on Clarkson's criteria, while the superscript notations ^∗^, ^∗∗^, and ^∗∗∗^ represent noncytotoxic, cytotoxic, and highly cytotoxic, respectively based on Meyer's criteria. ND: not determined. Aq. = aqueous extract; Me. = methanolic extract; Dc: dichloromethane extract.

## Data Availability

The data used to support the findings of this study are included within the article. Any additional data are available from the authors upon reasonable request.

## Supplementary Materials

Supplementary Materials A consent form and the questionnaire used in this study to collect ethnomedical data of plants used to manage SBE in Mwingi West Subcounty are presented in Appendices 1 and 2, respectively, in the additional file.
