# Supplementary material for: Ethnobotanical Documentation, Phytochemical Screening, and Cytotoxicity Evaluation of Medicinal Plants Used to Manage Snakebite Envenomation in Mwingi West Subcounty, Kenya
Source: Evid Based Complement Alternat Med. 2021 Sep 27;2021:4167296. doi: 10.1155/2021/4167296 (PMC8490041; doi:10.1155/2021/4167296)
Supplement: Supplementary Materials — A consent form and the questionnaire used in this study to collect ethnomedical data of plants used to manage SBE in Mwingi West Subcounty are presented in Appendices 1 and 2, respectively, in the additional file. [file 4167296.f1.docx]

**Appendix 1: CONSENT FORM**

**Title of the Study**: Ethnobotanical Documentation, Phytochemical Screening, and Cytotoxicity Evaluation of Medicinal Plants Used to Manage Snakebite Envenomation in Mwingi West Sub County, Kenya

**Principal investigator**: Stella Kwamboka Mokua

**Study Location**: Mwingi West Sub County, Kitui County, Kenya

**Purpose of the Study**: Academic (MSc. Thesis)

Dear Participant,

You have been selected to participate in this study. The study seeks; To help Ms. Stella Kwamboka Mokua, a postgraduate student undertaking an MSc. Degree in Pharmacology and Toxicology at the University of Nairobi.

To facilitate the collection of the relevant data, the investigator shall use a structured questionnaire containing questions about the plants you use or are used to manage snakebite envenomation (SBE) in this area, the parts used, modes of preparation, and routes of administration.

Confidentiality: The information you provide is confidential and will only be used for research purposes. Your participation is entirely voluntary, and you can withdraw from the study even after having agreed to participate.

Potential Benefits of the study: Ethnomedical documentation of plants used to manage snakebite envenomation for heritage and advancement of science.

Potential Risk of the Study: There is no risk associated with your participation in this study.

**Participant’s declaration**: I am a native resident/ herbalist of this region, have understood the purpose of this study, and am able to answer questions you have regarding the the use of traditional medicines used to manage snakebite envenomation.

Name:………………………………………………………..

*Signature: ………………………………………………………….*

*Contact information: ……………………………………………..*

*NB: If you have any questions about this study, you may ask me now or contact me via this address: P.O BOX 30197-00100, Nairobi: Phone Number: 0718312929*

**Appendix 2: ETHNOBOTANICAL DATA COLLECTION QUESTIONNAIRE**

**INSTRUCTIONS**

**Please fill in the blank spaces and tick in the appropriate Box**

**Personal Information of Respondents**

1. Name: …………………………………………………………………………………...
2. Gender:

Male

Female

1. Please specify your age category ………………………………………………..

20-35 years

36-50 years

50-65 years

65 and above

1. Education level: …………………………………………………………………

Primary

Secondary

Tertiary

Others specify

1. Source of income;

Employment

Business

Others, specify…………………………………………………………………

**Indicate Professional experience on the management of venomous bites**

1. Practice specification

Herbalists

Traditional practitioners

Local people

Others

1. Indicate years of experience? ……………………………………
2. What is the source of your knowledge? ……………………………………………...
3. Are you aware of Snakebite envenomation in this area?

Yes

No

1. Have you come across somebody bitten by a snake?

Yes

No

If yes, what plant (s) or combination do you use in managing the bite? …………………. ………………………………………………………………………………………………………………………………………………………………………………….

1. Where do you get the plant(s) from? ……………… …………………………………………………………………………………………...
2. Are the plants available?

Readily available

Scarcely available

Available in season:

Cold

Hot

1. Which part(s) of the plant did you use to prepare the remedy?

Leaves

Bark

Roots

Flowers

Pulp

Fruit

Seed

1. a) How do you prepare the herbal plant?

Dried

Fresh

Other……………………………………….……………………………

1. Please specify the mode of preparation of the herbal remedies …………………………………………………………………………………….…
2. Please indicate how the prepared drug is administered

Oral

Topical application

Others specify……………………………………………………………………

1. Indicate amount used ……………………………………………………………….
2. For how long should the patient take the drugs? ………………………………………………………………………
3. Do you give verbal instructions when administering this remedy?

Yes

No

1. Are the plant(s) used safely?

Yes

No

Participant declaration: I declare that I have answered the questions in this questionnaire to the honestly and to the best of my knowledge, and am available for further clarifications, should a need arise.

Signature………………………………………………..Name………………………………

Thank you.
